# The application of titanium dioxide (TiO_2_) nanoparticles in the photo-thermal therapy of melanoma cancer model

**DOI:** 10.22038/IJBMS.2018.30284.7304

**Published:** 2018-11

**Authors:** Mohammad Ali Behnam, Farzin Emami, Zahra Sobhani, Amir Reza Dehghanian

**Affiliations:** 1 Nano Opto-Electronic Research Center, Electrical and Electronics Engineering Department, Shiraz University of Technology, Shiraz, Iran; 2 Quality Control Department, Faculty of Pharmacy, Shiraz University of Medical Sciences, Shiraz, Iran; 3 Pharmaceutical Sciences Research Center, Shiraz University of Medical Sciences, Shiraz, Iran; 4 athology Department, Shiraz University of Medical Sciences, Shiraz, Iran

**Keywords:** Hyperthermia, Laser diode, Melanoma cancer, PEGylated titanium dioxide- (TiO_2_-PEG) nanoparticles

## Abstract

**Objective(s):**

Photo-thermal therapy (PTT) is a therapeutic method in which photon energy is converted into heat to induce hyperthermia in malignant tumor cells. In this method, energy conversion is performed by nanoparticles (NPs) to enhance induced heat efficacy. The low-cytotoxicity and high optical absorbance of NPs used in this technique are very important. In the present study, titanium dioxide (TiO_2_) NPs were used as agents for PTT. For increasing water dispersibility and biocompatibility, polyethylene glycol (PEG)-TiO_2_ NPs (PEGylated TiO_2_ NPs) were synthesized and the effect of these NPs on reducing melanoma tumor size after PTT was experimentally assessed.

**Materials and Methods:**

To improve the dispersibility of TiO_2_ NPs in water, PEG was used for wrapping the surface of TiO_2_ NPs. The formation of a thin layer of PEG around the TiO_2_ NPs was confirmed through thermo-gravimetric analysis and transmission electron microscopy techniques. Forty female cancerous mice were divided into four equal groups and received treatment with NPs and a laser diode (λ = 808 nm, P = 2 W & I = 2 W/cm^2^) for seven min once in the period of the treatment.

**Results:**

Compared to the mice receiving only the laser therapy, the average tumor size in the mice receiving TiO_2_-PEG NPs with laser excitation treatment sharply decreased.

**Conclusion:**

The results of animal studies showed that PEGylated TiO_2_ NPs were exceptionally potent in destroying solid tumors in the PTT technique.

## Introduction

Cancer still remains one of the leading causes of death with increasing incidence all over the world (1). The most pervasive method of cancer treatment is chemotherapy, which normally faces the problems of drug resistance and insufficient efficacy of drug delivery into cancer cells (1, 2). Another common method in cancer treatment is surgery. Methods of cancer treatment strongly rely on tumor size, lymph node involvement, and how much the cancer has spread. Surgery in combination with chemotherapy is the primary treatment for cancers (3, 4). The advent of nanoparticles (NPs) in biomedical and bioengineering fields made a revolution in the methods of cancer therapy. Nano-scale sizes of NPs improve their ability to be attached and transported to cells (2, 5). Nano-sized particles have been used in photodynamic therapy (PDT) and sonodynamic therapy of clinical cancer studies. PDT utilizes light absorbing photosensitizers to generate highly reactive oxygen species (ROS) that can cause cell rupture. Stimulated particles could fluctuate the electrons and have them transfer their charge from a state to another one, which produces active oxygen species (6). PDT has been used to treat malignant tumors and abnormal vasculatures (7). Production of toxic singlet oxygen and high photosensitivity of treated patients in this method could limit the PDT technique (4). Photo-thermal therapy (PTT) by means of NPs promises a new technique to efficiently treat cancer cells without any major limitation or side effects. In particular, NPs play an efficient role in converting the photon energy of laser light into heat due to their specific physicochemical properties and inducing hyperthermia in malignant tissues (2, 5). Thus far, a variety of nanostructures such as gold NPs (8), silver NPs (9), and carbon nanotubes (10) have been successfully developed in inducing hyperthermia in tumor tissues. A good candidate with specific characteristics for the PTT of tumors is titanium dioxide (TiO_2_) NPs.

Recently, TiO_2_ has attracted a growing deal of interest (11, 12). It is used in pharmaceutical and cosmetic industries, and is generally considered to be biologically inert (13). It is bio-friendly and has exceptional properties, such as high refractive index, and photocatalytic and magnetic properties (14–16)Such characteristics of TiO_2_ stem from the spontaneous formation of an oxide layer on the titanium surface (17). TiO_2 _can destroy bacteria, viruses, fungi, and cancer cells (18) and can act as an effective catalyzer for treating malignant tumors (4, 19). PDT, drug delivery, cell imaging, biosensors for biological assay, and genetic engineering are some forms of biomedical application of TiO_2_ NPs (5). TiO_2_ NPs could be a good choice for biomedical applications as agents in converting photon energy into heat in the PTT method, which is due to their super hydrophilicity (20), low-toxicity, good thermal conductivity, good optical absorption, and chemical and thermal stability *in vivo* (5). TiO_2_ nanostructures have been used in drug delivery systems for different anti-cancer drugs, such as daunorubicin, temozolomide, doxorubicin, and cisplatin (21–23). To increase the biocompatibility of TiO_2_ NPs, polyethylene glycol (PEG) could be attached to their surfaces. PEGylated NPs could escape the Reticulo-Endothelial System (RES) (18, 19). In the present study, TiO_2_ NPs were evaluated as impressive agents for PTT *in vivo*. To improve the dispersibility of TiO_2_ NPs, a layer of PEG coated the NPs. The efficacy of TiO_2_-PEG NPs in the treatment of melanoma cancer model in the PTT technique was also assessed.

## Materials and Methods

***Preparation and characterization of TiO***_2_ ***–PEG NPs***

The TiO_2_ NPs used in this study (particle size: 10–25 nm, purity: > 99%, phase: anatase) were purchased from US Research Nanomaterials, Inc., the United States. To enhance the dispersibility of the TiO_2_ NPs in deionized water, they were coated with a layer of PEG (10). Twenty five mg TiO_2_ NPs was suspended in 25 mL deionized water. Then, 250 mg PEG_1000_ (Sigma-Aldrich, St. Louis, MO, the USA) was dissolved in TiO_2_ NPs suspension. The suspension was ultrasonicated for 15 min and then stirred at room temperature overnight to allow the hydrophilic polymer to wrap around the TiO_2_ NPs (3). After being stirred, the suspension was centrifuged at 4000 rpm for 15 min to separate the unreacted TiO_2_ NPs, and afterward, the supernatant was collected (3, 10). 

The microscopic image of the TiO_2_-PEG NPs was taken by a transmission electron microscope (TEM) (Philips Electron Optics, the Netherlands). The light absorption spectrum of the TiO_2_ NPs was measured by a UV/Vis double beam spectrophotometer (PG Instruments Ltd., T80+ UV–Vis spectrophotometer, Lutterworth, the UK). Thermogravimetric analysis (TGA) was carried out by (METTLER TOLEDO, TGA_2_, Switzerland) under the dynamic atmosphere of an inert gas (N_2_) at 30 ml/min.

***Tumor induction***

All experimental standards of this study were endorsed by the Animal Care and Use Committee of Shiraz University of Medical Sciences, Shiraz, Iran, and the experiments were done in accordance with the National Institutes of Health Guidelines for Care and Use of Laboratory Animals. All procedures were verified to minimize discomfort to the animals and to use as few animals as possible for statistical analysis. Fortunately, this experiment was approved by the Ethical Committee at Shiraz University of Medical Sciences. 

A metastatic murine melanoma cell line, B16/F10 (NCBI C540 was purchased from the National Cell Bank of Pasteur Institute of Iran, Tehran, Iran) was cultured in an RPMI 1640 medium, under 5% CO_2_ at the temperature of 37 °C. It was then prepared by 10 % fetal bovine serum, 100 IU/mL of penicillin and 100 µg/mL streptomycin. Forty female C57BL/6J inbred mice, weighing 25–35 g, and aged 7–9 weeks were selected for the tumor induction. The murine melanoma cells at a number of 0.5 * 10^6^ were suspended in 200 µl culture medium and injected subcutaneously into the loose skin over the neck (10). The mice were housed in standard cages under standard conditions with 14:10 hr light/dark cycle (lights on at 6:00 a.m.), at an ambient temperature of 25 ± 2 °C and 30% relative humidity. They were randomly divided into four equal independent groups (N = 10) and had access to normal chow and water *ad libitum*. Melanoma is a superficial tumor, so its changes could be observed easily during the treatment. 

***Photo-thermal therapy of tumors***

Two weeks after the injection of the murine melanoma cell line, the melanoma tumors had sufficiently grown (approximately 1 cm^3^) to start the treatment. The animals were anesthetized by injecting Ketamine and Xylazine intramuscularly (IM). The tumor regions were shaved and measured by a caliper and an ultrasound machine (Ultrasonix SonixOP; Burnaby, BC, Canada). The tumor size was estimated through the following equation:

Tumor volume = (L/2)*W^2^ (mm^3^) (3, 10) 

 In this equation, L and W indicate the length and width of the tumor, respectively. The treatment started according to the following grouping: 

Group Ι (TiO_2_+laser): 200 µl/cm^3 ^(tumor volume) TiO_2_-PEG NPs (1 mg/ml) were injected directly into the tumor and then excited by a laser diode.

Group ΙΙ (Laser therapy): The laser therapy was done without any pre-treatment with the NPs.

Group ΙΙΙ (TiO_2 _NPs): 200 µl/cm^3 ^(tumor volume) TiO_2_-PEG NPs (1 mg/ml) were injected directly into the tumor without any laser excitation.

Group IV (Control): This group did not receive any treatment.

Groups Ι and ΙΙ were irradiated by a continuous wave (CW) near-infrared (NIR) laser diode (DAJCO, Shiraz, Iran) with these specifications: wavelength = 808 nm; power = 2 W; spot size = 1 cm^2^; and intensity = 2 W/cm^2^ for seven min once in the period of the treatment (10, 24). However, the control cases were not irradiated. The tumor sizes were measured three days after the laser excitation. After the period of the treatment, the animals were euthanized and their masses were excised for histopathologic examination (five cases of TiO_2_+laser group were euthanized after three months of follow-up). 

***Histopathological examination***

The specimens were treated, formalin fixed paraffin embedded (FFPE) blocks were provided, and the slides were stained with Hematoxylin and Eosin (H&E) method. The specimens were sampled for microscopy evaluation. 

***Statistical analysis***

The numerical results of this study were presented as mean ± standard deviation (SD). The normality of the results was analyzed by the one-sample Kolmogorov-Smirnov test. Significant differences between the values were statistically tested by Student’s t-test in each group. Multiple comparisons at multiple time points were tested by ANOVA with Repeated Measures. The statistical analyses were performed using SPSS® statistical software for Windows®, version 20.0 (SPSS Inc., Chicago, IL, USA). A *P*-value of < 0.05 was regarded as significant.

## Results

***Preparation and characterization of the TiO***_2_ ***-PEG NPs***

**Figure 1:**
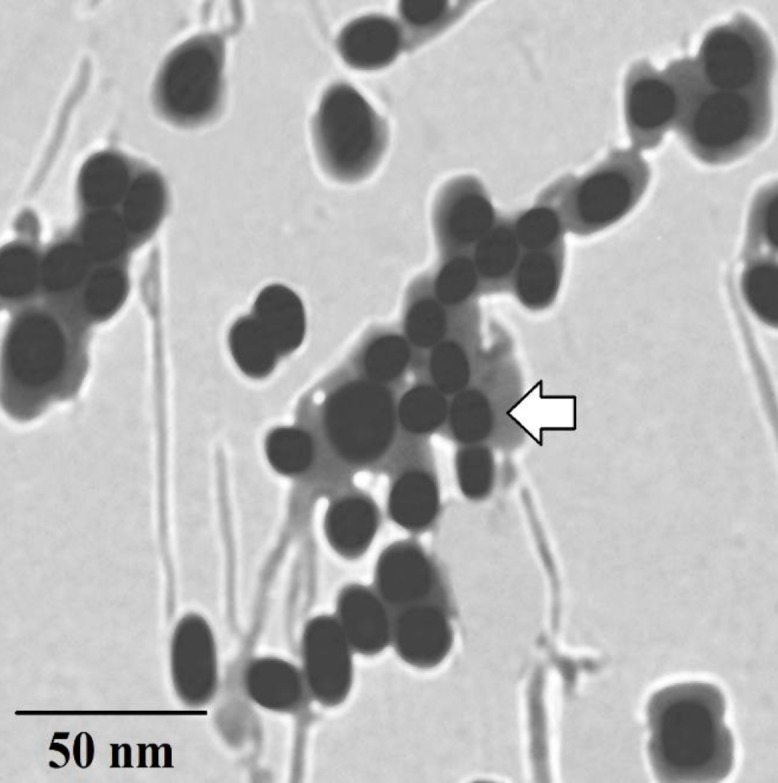
The TEM image of the TiO_2_-PEG NPs; a layer of PEG was formed on the surface of the TiO_2_ nanospheres (the arrow shows the PEG layer around the TiO_2_ NPs)

Figure 1 shows the TEM image of PEG-coated TiO_2_ NPs. Accordingly, a continuous layer of PEG with the average thickness of a few nanometers was formed on the surface of the TiO_2_ NPs.

**Figure 2:**
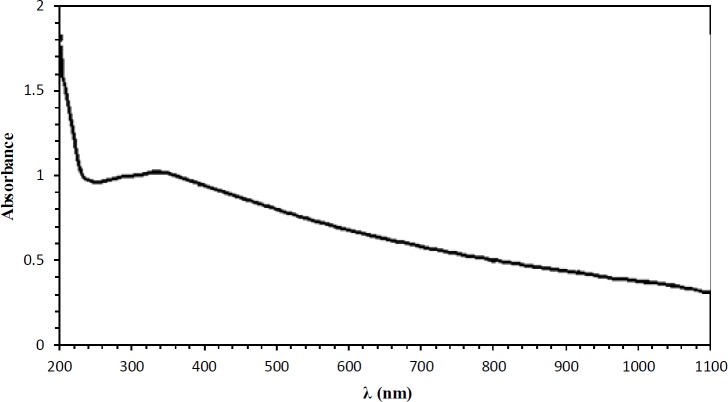
The UV-Vis light absorption spectrum of the TiO_2_-PEG NPs; absorption happened in two regions. It reached a peak at UV range, then gradually reduced at visible–near-IR range

The UV-Vis light absorption spectrum of the TiO_2_-PEG NPs is provided in Figure 2. As shown in this figure, absorption occurred in two regions. It reached a peak at the UV range, and then it gradually reduced at visible–near-IR range. Despite being at the wavelength of 808 nm, photoabsorption of these NPs is relatively low compared to those with a UV wavelength, but due to the deep penetration of NIR wavelength into the body (25), a CW NIR laser diode (808 nm) was used for photo irradiation (10). Additionally, the UV range is dangerous to the body and may cause gene mutation and DNA damage (26). 

**Figure 3:**
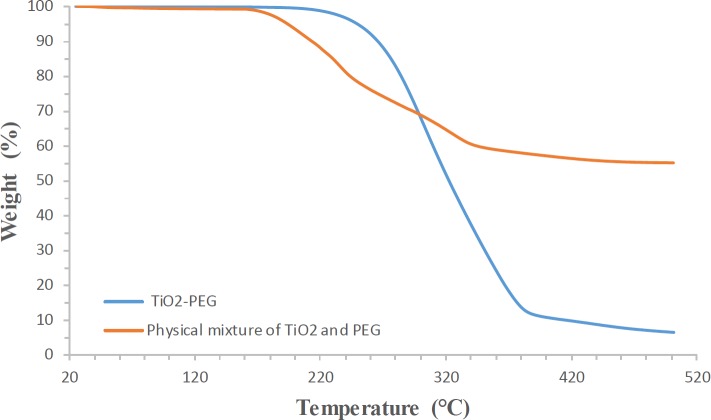
The thermo-grams of the physical mixture of the TiO_2_ NPs and PEG, and also synthesized TiO_2_-PEG; precisely, in the physical mixture of the TiO_2_ NPs and PEG, weight loss appeared at two stages: 224.16 °C and 299.26 °C. TiO_2_-PEG showed a completely different pattern

The TGA measurements provided further evidence regarding the interaction between the PEG and the surfaces of the TiO_2 _NPs. Figure 3 shows the thermogram of the physical mixture of the TiO_2_ NPs and PEG, and also the thermo-gram of TiO_2_-PEG. In the physical mixture of the TiO_2_ NPs and PEG, weight loss occurred at two stages, 224.16 °C and 299.26 °C. TiO_2_-PEG showed a completely different pattern in comparison with the physical mixture of the TiO_2_ NPs and PEG. In the TiO_2_-PEG pattern, weight loss occurred at one stage at 312.93 °C. 

**Figure 4:**
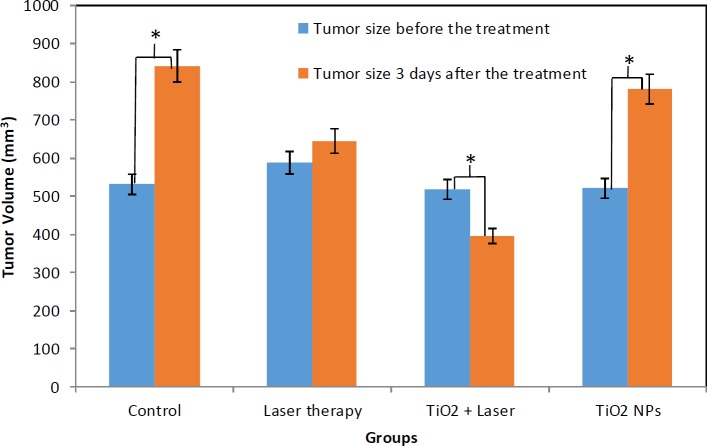
The tumor volume of different cases before and three days after PTT (* shows statistically significant difference (*P*<0.05)); the average tumor volume increased in the Control, TiO_2_ NPs, and Laser therapy groups, but it decreased in the TiO_2_+laser group

**Figure 5:**
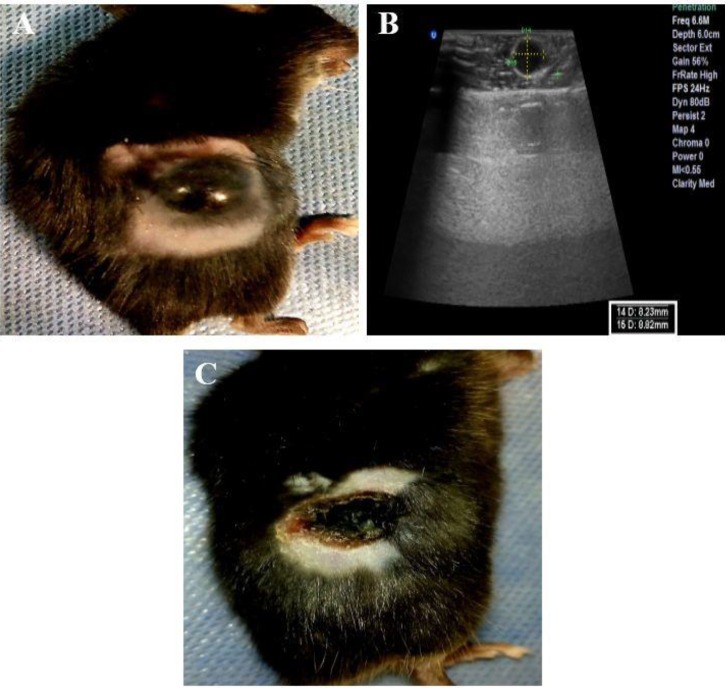
Tumor ablation stages in the TiO_2_+laser group with the PTT technique. (A, B): the photograph and the ultrasonography image of a cancerous mouse in the TiO_2_+laser group before the treatment, respectively. (C): the photograph of the mouse three days after the treatment (sonography was not feasible)

**Figure 6:**
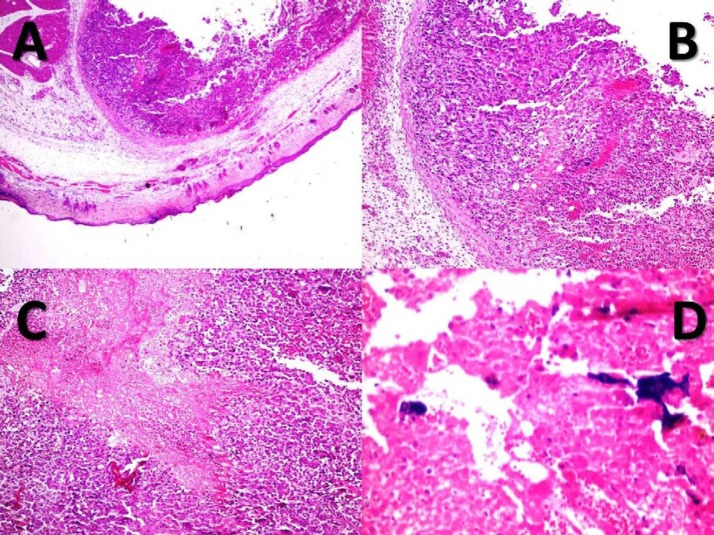
Nodular melanoma with a central necrosis in the TiO_2_+laser group. (A, B, C): histopathology of the skin (X40, H&E (A)), (X100, H&E (B, C)). (D): TiO_2_-PEG NPs present in the necrotic areas (X400, H&E). Accordingly, a severe necrosis was seen in the TiO_2_+laser group

**Figure 7:**
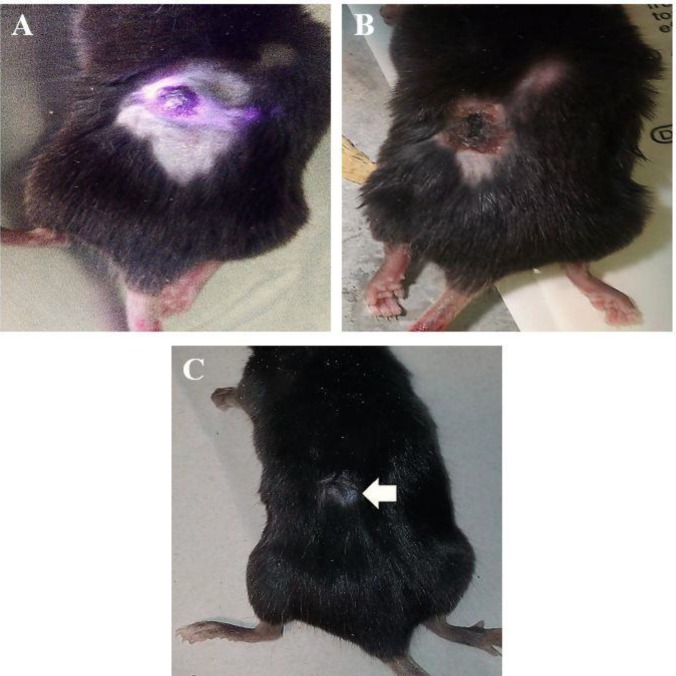
Three months of follow-up in the TiO_2_+laser cases treated with PTT. (A, B, C): before, three days, and three months after the treatment, respectively (the arrow indicates the changes of hair color in the tumor site after three months)

**Figure 8:**
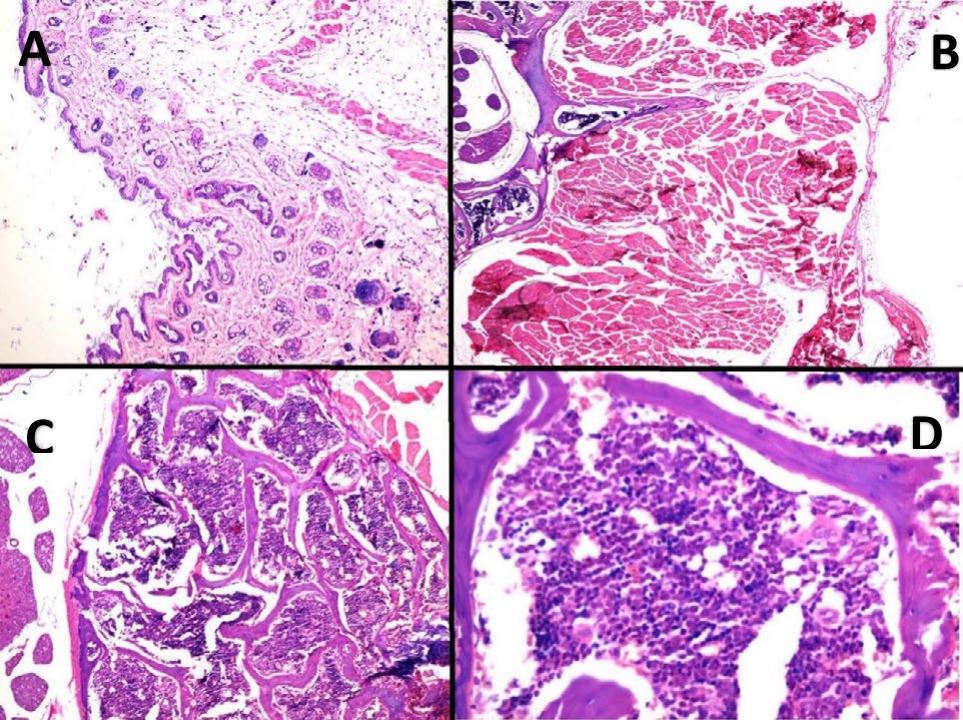
The histopathology of the treated cases in the TiO_2_+Laser group after three months of follow-up. (A): the histopathology of the skin shows no residue of the tumor and scar formation (X40, H&E). (B): the histopathology of the soft tissue of the back shows no evidence of tumor residue (X40). (C, D): the histopathology of the bone marrow shows normocellular marrow with polymorphic presence of hematopoietic cells (X40, X400)

**Table 1:** Histopathologic results of tumor tissues after treatment in different groups

Groups	Necrosis (%)	Breslow’s thickness	Tumor stage after the treatment according to AJCC 2010
TiO_2_+laser	(0.70±0.05)*100	**>4mm**	**IIA**
Laser therapy	(0.25±0.03)*100	>4mm	IIB
TiO_2_ NPs	< (0.05±0.01)*100	>4mm	IIB
Control	< (0.05±0.01)*100	>4mm	IIB

***Photo-thermal therapy of tumors***

The tumor sizes were recorded three days after laser excitation. The tumor sizes of different groups (before and three days after the treatment with PTT) were analyzed and the results showed significant differences between groups Ι, ΙΙΙ, and IV (*P*-value<0.05).

The decrease in tumor volume in group Ι is obvious as indicated in Figure 4. As can be seen in Figure 4, the average tumor volume in the Control and TiO_2_ NPs groups increased. However, it significantly decreased in the TiO_2_+laser group. The tumor sizes in the Laser therapy group were not significantly different during the treatment, but the tumors ceased to grow further. The slopes of tumor size against time in the Control, Laser therapy, TiO_2_+laser, and TiO_2 _NPs groups were about +103.58 mm^3^/days, +19.07 mm^3^/days, –40.60 mm^3^/days, and +86.70 mm^3^/days, respectively (“+” indicates increase and “–” indicates a decrease in tumor size).

The histopathologic evaluation of the tumors indicates a severe necrosis in the TiO_2_+laser group. Necrosis was the most important discriminator among the cases and its percentage was higher in the TiO_2_+laser group compared to the Laser therapy, TiO_2_, and Control groups, which indicates the noticeable effect of the TiO_2_-PEG NPs in inducing hyperthermia in the tumors when excited with a laser diode. Regressive fibrosis, lymphocytic infiltration, vascular invasion, or neurotropism were not seen in the cases. The histopathologic results are shown in Table 1 and Figure 6.

Five cases in the TiO_2_+laser group were followed up for three months after the treatment to analyze the trend of tissue regeneration. The stages of tumor treatment are shown in Figure 7, which indicates that the hair color of the tumor region changed after three months. 

For further examination, three months after the start of the treatment these cases were euthanized and their mass was formalin fixed and sent for histopathologic evaluation. As shown in Figure 8, histopathology of the skin, the soft tissue of the back, and the bone marrow of the treated cases showed that there was no evidence of melanoma cancer in TiO_2_+laser cases after three months of follow-up.

## Discussion

Titanium dioxide NPs have a wide variety of applications in medicine and life sciences. These NPs can be used as a carrier for drugs especially anti-cancer drugs and as an agent for photo-dynamic or PTT of solid tumors. TiO_2_ NPs have been used as drug delivery systems for different anti-cancer drugs, such as paclitaxel, doxorubicin, daunorubicin, temozolomide, and camptothecin (18, 27–30). In these studies, anti-tumor efficiency could be improved by TiO_2_ NPs. However, these NPs tend to aggregate in aqueous media and may cause problems in biological systems. In order to prevent the aggregation of these NPs, their surfaces should be modified. One of the most common methods to prevent aggregation of NPs is to cover them with hydrophilic polymers (31). PEG is one of the best polymers to solve this problem. Through attachment of PEG to the surface of the NPs, the biocompatibility of the NPs would be increased. In addition, PEGylated NPs evade the RES (18). Formation of a thin layer of polymer on the surface of TiO_2_ NPs through a simple adsorption method is reported in some studies (18, 32, 33). We used this polymer to improve the water dispersibility of TiO_2_ NPs. The TEM and TGA techniques indicated the formation of a thin layer around the NPs. Then, these prepared NPs were used for PTT of solid melanoma.

The photobiological effects of visible and NIR light rely on their wavelengths and could be affected by the structure, vasculature, thickness, and pigmentation of the skin’s strata (34, 35). It is reported that a polarized beam in the visible-NIR range can cause biological effects in cells through electron oscillation inducement (36). Optical radiation with a longer wavelength penetrates further into the body than a shorter one (25, 37). Visible light is widely absorbed by hemoglobin of the vasculature and the melanin located in the skin (37, 38). Infrared beam affects the body through transferring thermal energy into tissues. In the infrared spectrum, scattering increases largely in the body component ; therefore, light will penetrate deeply into the body (34, 39). NIR beam deeply penetrates into tissues and can be effectively utilized in cancer treatment. Some nano-structures, such as plasmonic NPs (40) and CNTs (10), can absorb NIR light and effectively convert its energy into heat. The stimulation of NPs causes vibrational stress of electrons and makes them transmit from the ground states to the excited states. The energy caused by electrons’ displacement is converted into heat by electron-electron relaxation and electron-photon relaxation (40, 41).

Stimulation of TiO_2_ NPs with electromagnetic radiation in the range of visible or NIR light causes generation of cytotoxic ROS that induce apoptosis along with increasing the tumor region temperature (42, 43). These effects are the principles of PDT and PTT, respectively. 

Many researchers showed phototoxic effect of TiO_2 _NPs after UV-A radiation on a series of cancer cell lines such as cervical cancer cells (HeLa), bladder cancer cells (T24), monocytic leukemia cells (U937), adenocarcinoma cells (SPC-A1), colon carcinoma cells (Ls-174-t), breast epithelial cancer cells (MCF-7, MDA-MB-468), glioma cells (U87), and human hepatoma cells (Bel 7402) (5).

Penetration of the UV-A spectrum in the body is too low. Besides, the UV-A spectrum could have an adverse effect on biological molecules. Therefore, we used NIR wavelength for tumor ablation. NIR wavelength is more transmissive through the body and has low attenuation in biological systems (44, 45). Wenjun Ni *et al.* showed that black TiO_2_ NPs are efficient as photosensitizers for PDT to kill bladder cancer cells. They used 808 nm light for irradiation of black TiO_2 _NPs (46). In reported studies, the efficacy of TiO_2_ NPs after UV and NIR irradiation on the cancer cells was evaluated in the *in vitro* cell culture medium (32, 47). Considering these studies, we performed our research in the *in vivo* melanoma tumor model. In order to assess the PTT effects of the TiO_2_-PEG NPs, after injection of the TiO_2_-PEG NPs into the tumor, tumor sites were irradiated by an 808 CW laser diode. Results showed that the localized NPs caused significant necrosis due to the deep penetration of NIR beam into the body and good photoabsorption of TiO_2_-PEG at the wavelength of 808 nm. The findings of the animal studies indicated that in the TiO_2_+laser group, not only did the tumor growth cease, but the tumor size also shrank. The histopathological examination showed 70% necrosis in the TiO_2_+laser group, which confirms the good efficiency of the TiO_2_-PEG NPs in the NIR spectrum for the PTT method. Furthermore, following up the TiO_2_+laser cases for three months demonstrates good biocompatibility of these NPs in this technique.

This reported application of the TiO_2_-PEG NPs for *in vivo* trials could promise a biocompatible agent for this cancer therapy technique. Considering the results, the next step is to assess the efficacy of the TiO_2 _NPs in tumor suppression by hyperthermia therapy and to deliver anti-cancer drugs to tumor sites, concurrently.

## Conclusion

The present study assessed the application of PEGylated TiO_2_ NPs in inducing hyperthermia and necrosis in malignant tumor cells for the PTT technique. The animal trials in this study confirmed the relatively high efficacy of such NPs in destroying solid tumors without any symptom of cancer cells in treated cases. Therefore, the TiO_2_-PEG NPs could be utilized as a potent agent with low toxicity in the PTT technique for ablating solid tumors. 
